# The Efficacy and Safety of SGLT2 Inhibitors in Diabetes Kidney Transplant Recipients: A Systematic Review and Meta-Analysis

**DOI:** 10.12688/f1000research.162502.1

**Published:** 2025-03-25

**Authors:** Kanachai Boonpiraks, Pajaree Krisanapan, Suthiya Anumas, Charat Thongprayoon, Wisit Cheungpasitporn, Pattharawin Pattharanitima

**Affiliations:** 1 Chulabhorn International College of Medicine, Klong Luang, Pathum Thani, 12120, Thailand; 2 Internal Medicine, Thammasat University, Klong Luang, Pathum Thani, 12120, Thailand; 3 Department of Medicine, Mayo Clinic Minnesota, Rochester, Minnesota, 55905, USA

**Keywords:** SGLT-2 inhibitors, diabetes mellitus, kidney transplant

## Abstract

**Background:**

Sodium-glucose cotransporter 2 (SGLT-2) inhibitors have shown cardiorenal benefits in the general population; however, evidence regarding their efficacy and safety in kidney transplant recipients (KTRs) remains sparse. This meta-analysis seeks to evaluate the therapeutic efficacy and safety profile of SGLT-2 inhibitors specifically in the diabetes KTR population.

**Methods:**

We conducted a systemic review and meta-analysis following a registered protocol in the PROSPERO (CRD42023404886). A comprehensive literature search was performed using PubMed, Cochrane, and Scopus databases up to October 23 ^th^, 2024. We included observational and clinical trials which compared SGLT-2 inhibitors with control groups in KTRs. Risk of bias was evaluated by funnel plot. We reported differences in treatment effects as risk ratios (RRs) or weighted mean difference (WMD) with 95% confidence intervals (CIs).

**Results:**

A total of seven studies comprising 2,713 patients were included in this analysis. SGLT-2 inhibitors were associated with a significant reduction in HbA1c levels (WMD -0.37%; 95% CI -0.73 to -0.01; p = 0.04) and BMI (WMD -0.89 kg/m ^2^; 95% CI -1.27 to -0.50; p < 0.001). Additionally, SGLT-2 inhibitors demonstrated a beneficial effect in reducing mortality (RR 0.25; 95% CI 0.06 to 0.98; p = 0.05) and cardiovascular disease (CVD) (RR 0.41; 95% CI 0.17 to 0.98; p = 0.04). However, SGLT-2 inhibitors did not demonstrate benefit in kidney-related outcomes. Importantly, there was no significant increase in adverse events, including urinary tract infections, genital mycotic infections, urosepsis, or allograft rejection.

**Conclusions:**

SGLT-2 inhibitors effectively reduced mortality, cardiovascular disease, HbA1c levels and BMI in KTRs without increasing the risk of infections or allograft rejection. However, they did not demonstrate a significant benefit in kidney-related outcomes.

## Introduction

Diabetes mellitus (DM) and post-transplantation DM (PTDM) is a well-recognized transplant comorbidity. Despite the distinct pathophysiological mechanisms, both conditions contribute to poorer prognosis and decreased survival rates in kidney transplantation recipients (KTRs). ^1, 2^

A recent meta-analysis indicated a significant increase in all-cause mortality and graft failure in PTDM patients with hazard ratios (HR) of 1.67 and 1.35, respectively. ^3^ Moreover, transplant recipients carry a significantly higher cardiovascular (CV) risks compared to the general population. ^4, 5^ This risk is further heightened with comorbid pretransplant DM or PTDM. ^6, 7^ For instance, a long-term study in KTRs reported that PTDM and pretransplant DM were associated with a 1.2-fold and 5.1-fold increased risk of all-cause mortality, respectively, with CV events being the leading cause of death. ^7^ Therefore, in order to improve outcomes among kidney transplant patients, it is crucial to prioritize proper glycemic control and reduce CV risks.

Sodium-glucose cotransporter 2 (SGLT-2) inhibitors demonstrate substantial potential in mitigating cardiovascular risks and improving long-term renal outcomes. ^8, 9^ The EMPA-REG OUTCOME trial first reported the CV benefits of Empagliflozin in type 2 diabetic mellitus (T2DM) patients, demonstrating a notable decrease in CV death. ^10^ Subsequently, there has been a surge of interest in SGLT-2 inhibitors. Several meta-analyses have consistently demonstrated a reduction in the risk of major adverse cardiovascular events (MACE), including non-fatal myocardial infarction (MI), non-fatal stroke and CV deaths as well as a reduction in hospitalized heart failure. ^11, 12^

In patients with chronic kidney disease (CKD), strong evidence from large randomized controlled trials (RCTs) has consistently shown that SGLT-2 inhibitors can significantly reduce kidney disease progression and cardiac death by 28-39%. ^20– 22^ These reno-protective effects are particularly notable in patients, irrespective of coexisting type 2 diabetes (T2DM). ^9^ Nevertheless, there remains a paucity of large-scale randomized controlled trials (RCTs) and comprehensive meta-analyses to conclusively validate the efficacy and safety profile of SGLT-2 inhibitors in KTRs. As a result, clinical guidelines for the administration of SGLT-2 inhibitors in this specific population are currently constrained.

A recent meta-analysis ^13^ performed in 2020, comprising 8 studies involving a total of 132 participants, has provided evidence of the effectiveness of SGLT-2 inhibitors in treating DM among KTRs. The analysis demonstrated that SGLT-2 inhibitors effectively lower HbA1c levels, reduce body weight, and preserve kidney function, without reporting any serious adverse events. Previous research has primarily concentrated on evaluating differences between baseline and study endpoint measures, rather than employing a comparative analysis with a control group. Thus, we conducted this meta-analysis to compare the outcomes of SGLT-2 inhibitors with control groups. The aim of this study is to investigate the efficacy of SGLT-2 inhibitors in terms of renal benefits, glycemic control, metabolic profile improvement, mortality, and CVD outcome, as well as the safety profiles of SGLT-2 inhibitors among KTRs.

## Methods

### Search strategy and selection criteria

The meta-analysis was conducted following a registered protocol in the International Prospective Register of Systematic Reviews (PROSPERO; CRD42023404886). A systematic literature search of PubMed, Cochrane and Scopus databases from the inception until October 23, 2024 was conducted independently by three investigators (K.B., P.K. and P.P.). The search terms, including “Kidney” AND “Transplant” AND “Sodium glucose co-transporter 2 inhibitor” were used to assess the efficacy and safety of SGLT-2 inhibitors among kidney transplant recipients. Supplement material **(**Supplement; Table S1 **)** provides a comprehensive description of the search strategy employed. The search was limited to human studies, and no language restrictions were applied. Additionally, a manual search of the references of the included studies was conducted for additional relevant studies. The implementation and reporting of this meta-analysis adhered to the standards of the PRISMA Statement. ^14^

Studies were included in this meta-analysis if they were observational (case-control or cohort) or clinical trials that evaluated cardiovascular and kidney outcomes of SGLT-2 inhibitors compared to a control group in adults aged 18 years or older who had undergone kidney transplantation. Eligible studies had to report alterations in at least one of the following outcomes: estimated glomerular filtration rate (eGFR), urine protein creatinine ratio (UPCR), hemoglobin A1c (HbA1c), systolic blood pressure (SBP), body mass index (BMI), and adverse outcomes. Studies that primarily reported other outcomes or that included other glucose lowering medications without a subgroup report for SGLT-2 inhibitors alone were excluded. Retrieved studies were independently reviewed for eligibility by three investigators (K.B., S.A. and P.P.). Discrepancies were resolved through discussion among all authors.

### Data extraction and quality assessment

Data collection was done independently by three investigators (K.B., S.A. and P.P). A standardized data collection form was used to collect the following variables from each included study: study title, author names, publication year, study type, the country where the study was conducted, study drugs, number of participants, age, gender, type of DM (T2DM vs PTDM), transplant duration, immunosuppressive drugs, follow-up time, eGFR, UPCR, HbA1c, BMI, SBP, DBP, and adverse events.

The quality of each study was independently assessed by each investigator (P.P. and P.K.). Randomized controlled trials (RCTs) were evaluated using the Cochrane Risk of Bias (RoB 2) tool ^15^ (Supplement; Table S2), while non-randomized control studies were evaluated by the Risk of Bias In Non-randomized Studies – of Interventions (ROBIN-I) tool ^16^ (Supplement; Table S3). Publication bias was examined by a funnel plot.

### Statistical analysis

This meta-analysis was conducted using Review Manager (RevMan) [Computer program] Version 5.4. The Cochrane Collaboration, 2020. The differences in effects were reported as risk ratios (RRs) with 95% confidence intervals (CIs) for dichotomous outcomes and the weighted mean difference (WMD) with 95% CIs for continuous outcomes. In cases where the required data was not originally provided in the articles, we procured it through direct communication with the investigators. Participants in RCTs were analyzed in intention-to-treat groups.

Heterogeneity was evaluated using χ ^2^ and/or the I ^2^ statistic, where an I ^2^ value above 50% or a p-value below 0.1 indicated significant heterogeneity. In cases of significant heterogeneity, a random-effect model was employed for the meta-analysis. Publication bias was assessed by funnel plot. ^17^. Planned subgroup analysis for the primary outcomes stratified by the types of publication (observational studies vs. controlled trials) was not performed due to insufficient data. For all analyses, statistical significance was defined as a p-value less than 0.05.

## Results

### Study characteristics

**Figure 1.**
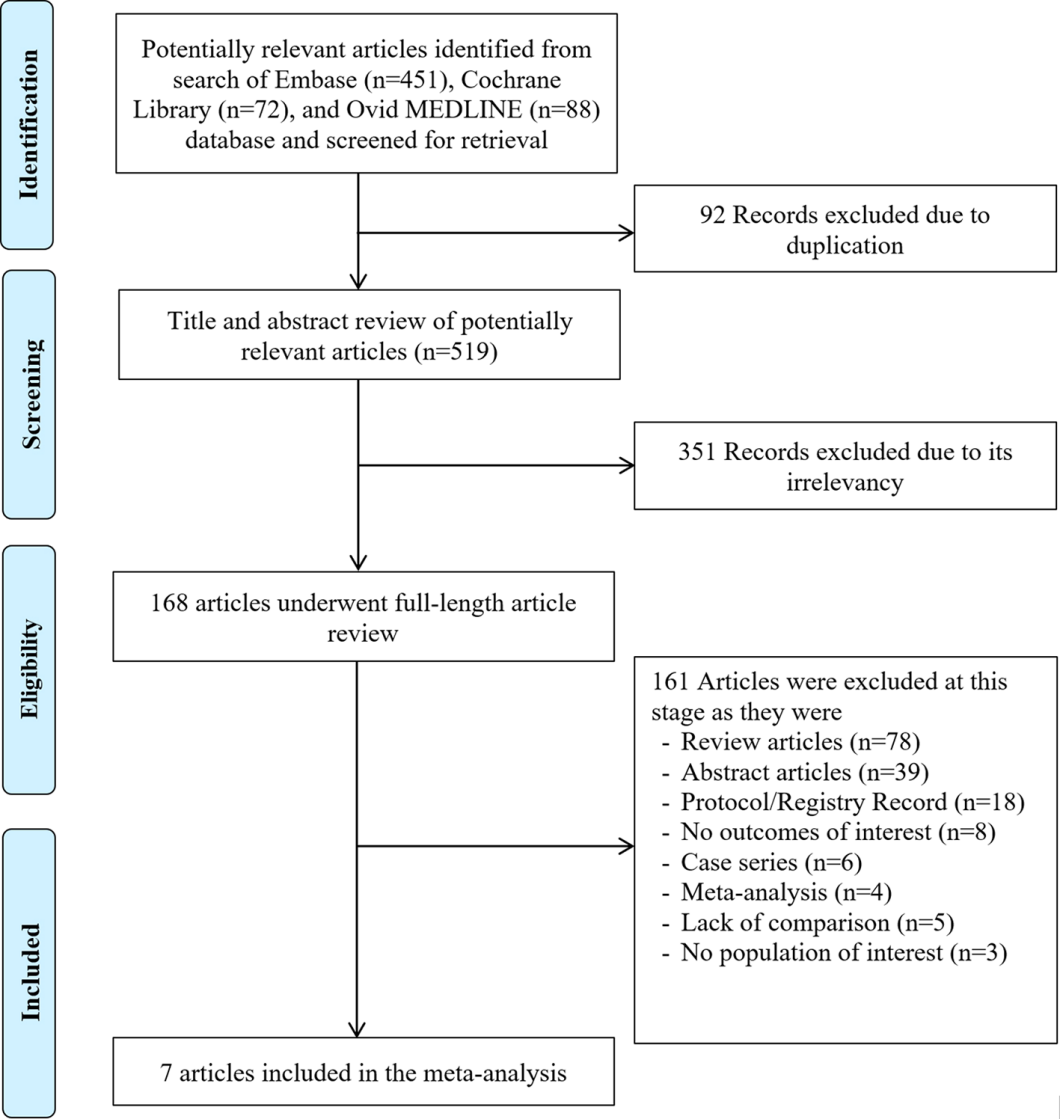
PRISMA flow of search methodology and selection process.

Our search strategy identified a total of 611 potential articles as shown in Figure 1, with 92 duplicates identified and subsequently removed. The remaining 519 articles underwent an initial screening based on their titles and abstracts, leading to the exclusion of 351 articles due to their lack of relevance. Consequently, 168 articles underwent a full comprehensive review. 161 studies were excluded based on factors such as publication type, lack of outcomes of interest, inappropriate population of interest, ongoing trials, and full-text unavailable. As a result, a total of 7 studies were included in this meta-analysis, consisting of 5 retrospective cohort studies, ^17– 21^ 1 RCT, ^22^ and 1 prospective interventional trial, ^23^ with baseline characteristics summarized as shown in Table 1.

**Table 1.** Characteristics of the Included Studies.

Author (Year)	Halden et al. (2019)	Schwaiger et al. (2019)	Hisadome et al. (2021)	Lim et al. (2022)	Demir et al. (2023)	Mahmoud et al. (2023)	Lim et al. (2024)
**Study type**	Double-blinded, randomized controlled trial	Prospective, interventional trial (with 2 phases of insulin and a switch to SGLT2i)	Retrospective cohort with IPTW analysis	Retrospective cohort with propensity score matching	Retrospective cohort	Retrospective cohort with case-matching	Retrospective cohort with propensity score matching
**Country**	Norway	Austria	Japan	Korea	Türkiye	Kuwait	Korea
**Total participants, n**	44	14 ^a^	85	2083	57	168 ^f^	254
**SGLT2i**	22	8	28	226	36	98	127
**Control**	22	14	57	1857	21	70	127
**Study drug(s)**	Empagliflozin	Empagliflozin	Various SGLT2i ^b^	Empagliflozin or dapagliflozin	Empagliflozin or dapagliflozin	Canagliflozin	Empagliflozin or dapagliflozin
**Control(s)**	Placebo	Insulin	Other oral hypoglycemic agents ^c^	Metformin or DDP-4 inhibitors or insulin	Other antiglycemic therapies, including insulin	Metformin, sulfonylurea, DPP4 inhibitors or insulin	Metformin, Sulfonylurea, DPP4 inhibitors or insulin
**Total follow-up duration, months**	6	12	12	9 ^e^	12	12	56.3
**Type of DM, n (%)**							
**PTDM**	44 (100)	14 (100)	0	475 (22.8)	31 (54.4)	75 (44.6)	43 (16.9)
**T2DM**	0	0	85 (100)	1608 (77.2)	26 (45.6)	93 (55.4)	211 (83.1)
**Duration of DM, years**	N/A	5.7 ± 4.8	N/A	N/A	14.8 ± 11.0	N/A	N/A
**Male, n (%)**	34 (77.3)	7 (50)	65 (76.5)	1399 (67.2)	36 (63.2)	107 (63.7)	179 (70.5)
**Age, years**	*SGLT-2i*: 63 [31, 72] *Control*: 59 [21,75]	56.5 ± 7.9	55.4 ± 8.7	52.4 ± 10.7	51.3 ± 11.0	*SGLT-2i*: 56 [N/A] *Control*: 57 [N/A]	*SGLT-2i*: 54 [46, 60] *Control*: 55 [47, 60]
**Weight, kg**	*SGLT-2i*: 92 [81.8, 104.5] *Control*: 84 [79.3, 94.0]	74.8 ± 17.2	71.0 ± 10.4	N/A	N/A	N/A	N/A
**BMI, kg/m** ^**2**^	*SGLT-2i*: 28.8 [24.7, 39.3] *Control*: 27.5 [22.4, 45.8]	27.3 ± 5.2	25.7 ± 3.6	23.9 ± 3.6	28.5 ± 4.5	*SGLT-2i*: 31.4 [N/A] *Control*: 29.7 [N/A]	*SGLT-2i*: 25.3 [22.0, 27.4] *Control*: 25.2 [23.1, 27.7]
**History of CAD, n (%)**	N/A	10 (71)	43 (50.6)	253 (12.1)	27 (47.4)	N/A	33 (13.0)
**Systolic BP, mmHg**	*SGLT-2i*: 143 [111, 176] *Control*: 140 [100, 163]	150 ± 26	128 ± 15	N/A	N/A	N/A	N/A
**Diastolic BP, mmHg**	*SGLT-2i*: 79 [63, 94] *Control*: 82 [55, 94]	86 ± 14	73 ± 10	N/A	N/A	N/A	N/A
**eGFR, ml/min/1.73m** ^**2**^	*SGLT-2i*: 66 [41, 83] *Control*: 59 [44, 82]	55.6 ± 20.3	48.5 ± 13.4	68.2 ± 19.9	72.4 ± 18.7	*SGLT-2i*: 67.2 [N/A] *Control*: 63.8 [N/A]	N/A
**Creatinine, mg/dL**	N/A	1.3 ± 0.4	N/A	1.2 ± 0.5	1.1 ± 0.3	N/A	N/A
**UPCR (mg/g)**	*SGLT-2i*: 100 [60, 150] *Control*: 90 [50, 120]	289 [190-808]	400 ± 600	N/A	*SGLT-2i*: 321 [45, 2565] *Control*: 229 [63, 909]	N/A	N/A
**Fasting blood sugar (mg/dL)**	*SGLT-2i*: 144 [90, 236] *Control*: 132 [81, 225]	N/A	N/A	143 ± 67	N/A	N/A	N/A
**HbA1c (%)**	*SGLT-2i*: 6.9 [6.5, 8.2] *Control*: 6.8 [6.1, 7.2]	6.5 ± 0.8	7.6 ± 1.2	7.3 ± 1.4	8.1 ± 1.9	*SGLT-2i*: 8.0 [N/A] *Control*: 7.2 [N/A]	*SGLT-2i*: 7.1 [6.3, 8.1] *Control*: 6.9 [6.1, 7.8]
**Hemoglobin (g/dL)**	*SGLT-2i*: 13.9 [13.1, 14.4] *Control*: 13.2 [12.1, 14.6]	12.7 ± 1.9	N/A	N/A	N/A	N/A	N/A
**Hematocrit (%)**	*SGLT-2i*: 43 [39, 45] *Control*: 43 [39, 44]	38.8 ± 5.4	N/A	N/A	N/A	N/A	N/A
**Other glucose-lowering therapy, n (%)**							
**Insulin**	8 (18.2)	14 (100)	61 (71.8)	1155 (55.4)	29 (50.9) ^d^	102 (60.7)	N/A
**Metformin**	2 (4.5)	N/A	4 (4.7) ^d^	1224 (58.8)	N/A	136 (81)	N/A
**Sulfonylurea**	7 (15.9)	N/A	4 (4.7) ^d^	725 (34.8)	N/A	16 (9.5)	N/A
**DPP4 inhibitors**	N/A	N/A	42 (49.4) ^d^	1144 (54.9)	N/A	66 (39.3)	N/A
**Time Since Transplant, years**	*SGLT-2i*: 3 [1, 16] *Control*: 3 [1, 15]	5.8 ± 4.8	N/A	0.25	*SGLT-2i*: 5 [0, 13] *Control*: 1.5 [0, 9]	7.4 ± N/A	1.12 [0.2, 2.51]
**Living donor, n (%)**	16 (36)	N/A	N/A	1483 (71.2)	54 (94.7)	127 (75.6)	200 (78.7)
**Immunosuppressive drugs, n (%)**							
**Tacrolimus**	35 (79.5)	11 (79)	83 (97.6)	1705 (81.9)	57 (100)	127 (75.6)	221 (87.0)
**Cyclosporin**	6 (13.6)	3 (21)	3 (3.5)	406 (19.5)	N/A	31 (18.5)	36 (14.2)
**Everolimus**	2 (4.5)	0	25 (29.4)	N/A	N/A	N/A	N/A
**Mycophenolate**	40 (90.9)	14 (100)	61 (71.8)	N/A	57 (100)	N/A	N/A
**Steroid**	43 (97.7)	14 (100)	85 (100)	2038 (97.9)	57 (100)	N/A	N/A
**Trough level, ng/mL**							
**Tacrolimus**	*SGLT-2i*: 5.4 [4.6, 6.9] *Control*: 6.2 [5.0, 6.8]	8.4 ± 3.1	2.0 ± 1.1	6.0 ± 1.5	N/A	N/A	N/A
**Cyclosporin**	*SGLT-2i*: 94 [80, 105] *Control*: 100 [94, 100]	64.0 ± 11.5	N/A	N/A	N/A	N/A	N/A
**Everolimus**	*SGLT-2i*: 6.2 [6.2, 6.2] *Control*: 10 [10, 10]	N/A	N/A	N/A	N/A	N/A	N/A
**Source of funding**	South-Eastern Norway Regional Health Authority, the Norwegian Diabetes Association, and Oslo Diabetes Research Centre.	None	None	Ministry of Health & Welfare, Republic of Korea and the Korean Society of Nephrology	None	None	Ministry of Health & Welfare, Republic of Korea and the Ministry of Education

**Abbreviations:** BP, blood pressure; CAD, coronary artery disease; DPP4, dipeptidyl peptidase 4; GLP-1RAs, Glucagon-Like Peptide-1 Receptor Agonists; IPTW, inverse probability of treatment weighting analysis; N/A, no data available; OADs, oral antidiabetic drugs; PTDM, post-transplant diabetic mellitus; SGLT-2i, sodium-glucose cotransporter 2 inhibitors; T2DM, type 2 diabetes mellitus; UK, United Kingdom; UPCR, urine protein creatinine ratio; USA, United States of America

^a^ Started with 14 patients but completed study with only 8

^b^ Canagliflozin or Ipragliflozin or Luseogliflozin or Empagliflozin or Dapagliflozin or Tofogliflozin

^c^ DDP-4 inhibitors or Glinides or Metformin or Sulfonylureas or Alpha-glucosidase inhibitors-1

^d^ Exclusively in the control group

^e^ Total follow-up time was 62.9 ± 42.2 months but the efficacy outcomes were reported after 9 months from the baseline

^f^ Counted only patients received SGLT2i and the control group

Overall, a total of 2,713 patients were included in this systematic review, with 545 patients in the SGLT-2 inhibitors group. These agents were primarily indicated for T2DM (74%) more than PTDM (26%). Specific SGLT-2 inhibitors prescription was reported in 383 participants from 5 studies, ^17– 19, 22, 23^ empagliflozin was the most frequently administered, accounting for 48%, followed by canagliflozin (28%), dapagliflozin (20.6%), ipragliflozin (1.8%), luseogliflozin (1.3%), and tofogliflozin (0.3%). In the control group, only one RCT ^22^ used a placebo, while the remaining studies ^17– 21, 23^ utilized other hypoglycemic agents, including insulin, metformin, dipeptidyl peptidase 4 (DPP-4) inhibitors, and sulfonylurea. Insulin was prescribed for 13.4% of 2,451 patients, whereas metformin was the most common oral hypoglycemic agent (OHA), accounting for 57% of 2,380 patients evaluated, ^18– 20, 22^ followed by DPP-4 inhibitors, which accounted for 54% of 1,252 patients evaluated. ^18– 20^

The median total follow-up duration was 9 [9, 12] months. Most patients were male (67%) with a mean age of 53.1 ± 11.7 years and a BMI of 24.7 ± 4.3 kg/m ^2^. The baseline HbA1c was 7.3 ± 1.4, and the duration of DM was reported in only 2 studies ^17, 23^ with a mean of 13.0 ± 10.1 years among 99 patients evaluated. Notably, only 15% of the 2,501 patients evaluated ^17– 19, 21, 23^ had a history of coronary artery disease (CAD).

In terms of baseline allograft function, the mean serum creatinine (Cr) was 1.2 ± 0.5 mg/dL among 2,154 evaluated patients, ^17, 19, 23^ and the mean eGFR was 67 ± 21 ml/min/1.73 m ^2^ among 2,451 evaluated patients. Of the 200 available patients, the mean UPCR was 293 ± 919 mg/g of Cr. ^17, 18, 22, 23^ The time since transplant ranged from 3 months to 7.4 years with a median duration of 1.8 [0.3, 5.6] years. Nearly two-thirds (77%) of 2,454 reported patients received kidneys from living-donors. ^17, 19– 22^ The most predominant immunosuppressive medication was tacrolimus. Among 2,226 patients, the tacrolimus trough level was 5.9 ± 1.7 ng/mL. ^18, 19, 22, 23^

### Efficacy of SGLT-2 inhibitors on kidney-related outcomes

**Figure 2.**
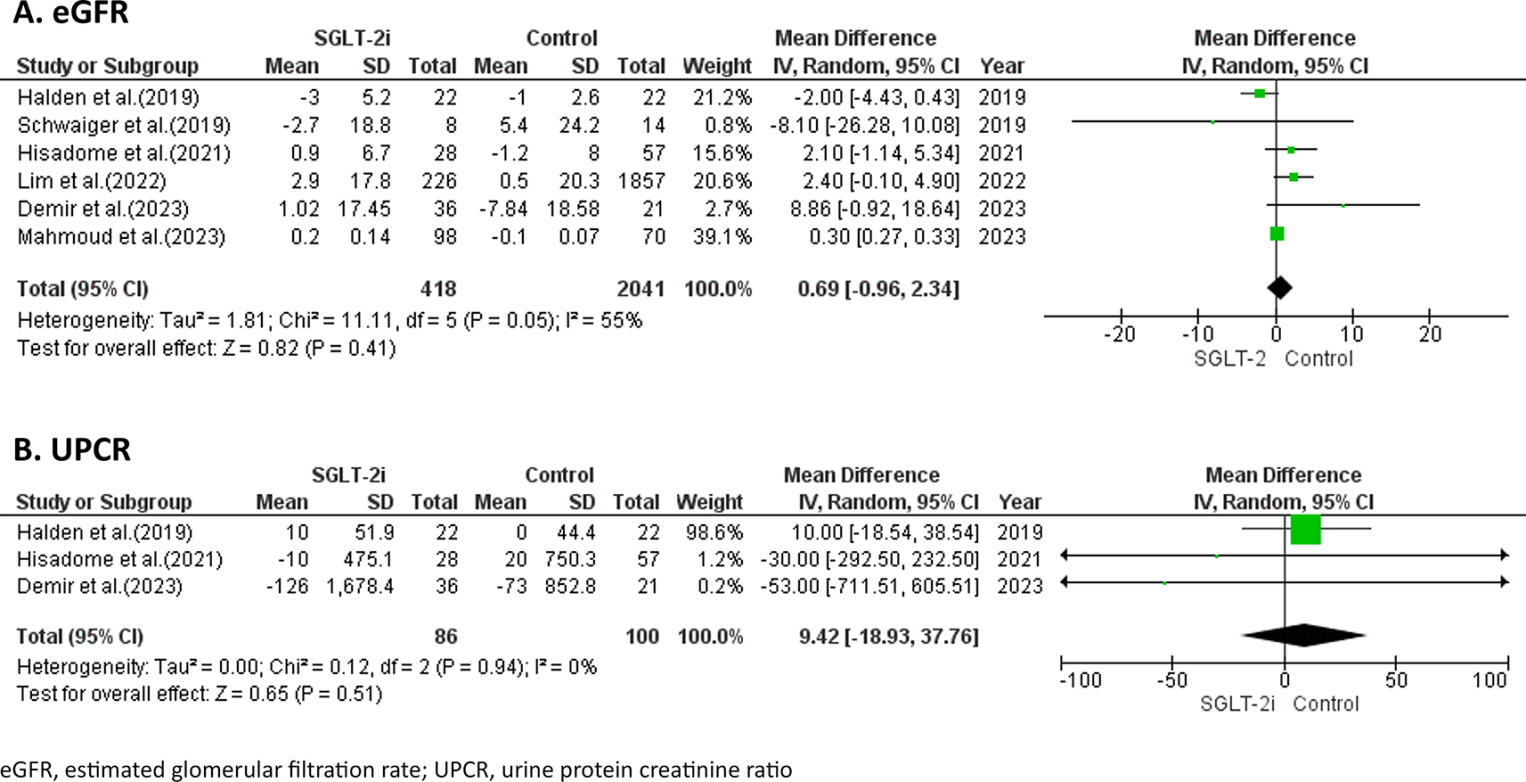
Forest plots of the efficacy of SGLT-2 inhibitors on kidney-related outcomes.

Kidney related outcomes were evaluated based on changes in eGFR and reductions in UPCR from baseline. Among 6 studies, ^17– 20, 22, 23^ the use of SGLT-2 inhibitors did not result in a significantly higher eGFR compared to the control, with a weighted median difference (WMD) of 0.69 mL/min/1.73 m ^2^ (95%CI -0.96, 2.34; *p* = 0.41; I ^2^ = 55%) as shown in Figure 2A. In terms of UPCR reduction, only 3 studies ^17, 18, 22^ involving 186 patients reported no statistically significant decrease in UPCR during the follow-up period (WMD = 9.42 mg/g; 95%CI -18.93, 37.76; *p* =0.51, I ^2^ = 0%) as shown in Figure 2B.

### Efficacy of SGLT-2 inhibitors on metabolic profiles

**Figure 3.**
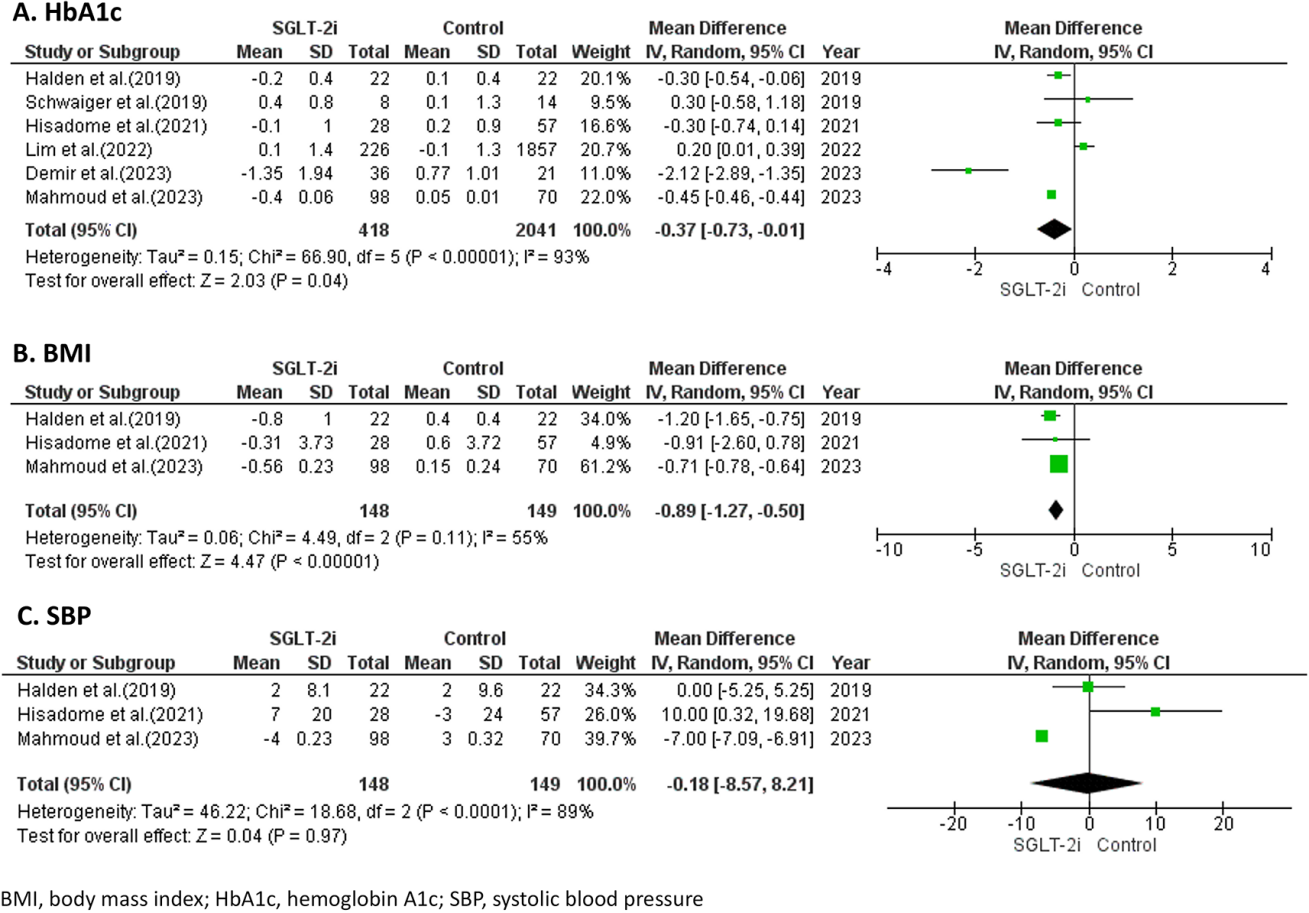
Forest plots of the efficacy of SGLT-2 inhibitors on metabolic profiles.

Across 6 studies, ^17– 20, 22, 23^ SGLT-2 inhibitors resulted in a significant reduction in HbA1c levels by -0.37% (95% CI -0.73, -0.01; p = 0.04) compared with the control, with significant heterogeneity (I ^2^ = 93%) as shown in Figure 3A. Additionally, SGLT-2 inhibitors significantly reduced BMI from baseline compared with the control, with a WMD of -0.89 kg/m ^2^ (95% CI -1.27, -0.50, p <0.001, I ^2^ = 55%) as shown in Figure 3B.

However, regarding the effect on blood pressure, data on systolic blood pressure (SBP) were reported in only 3 studies ^18, 20, 22^ involving a total of 297 patients. The analysis revealed no significant reduction in SBP with SGLT-2 inhibitors compared to the control, with a WMD of -0.18 mmHg (95%CI -8.57, 8.21; p = 0.97), with significant heterogeneity (I ^2^ = 89%) as shown in Figure 3C.

### Efficacy of SGLT-2 inhibitors on all-cause mortality and cardiovascular diseases

**Figure 4.**
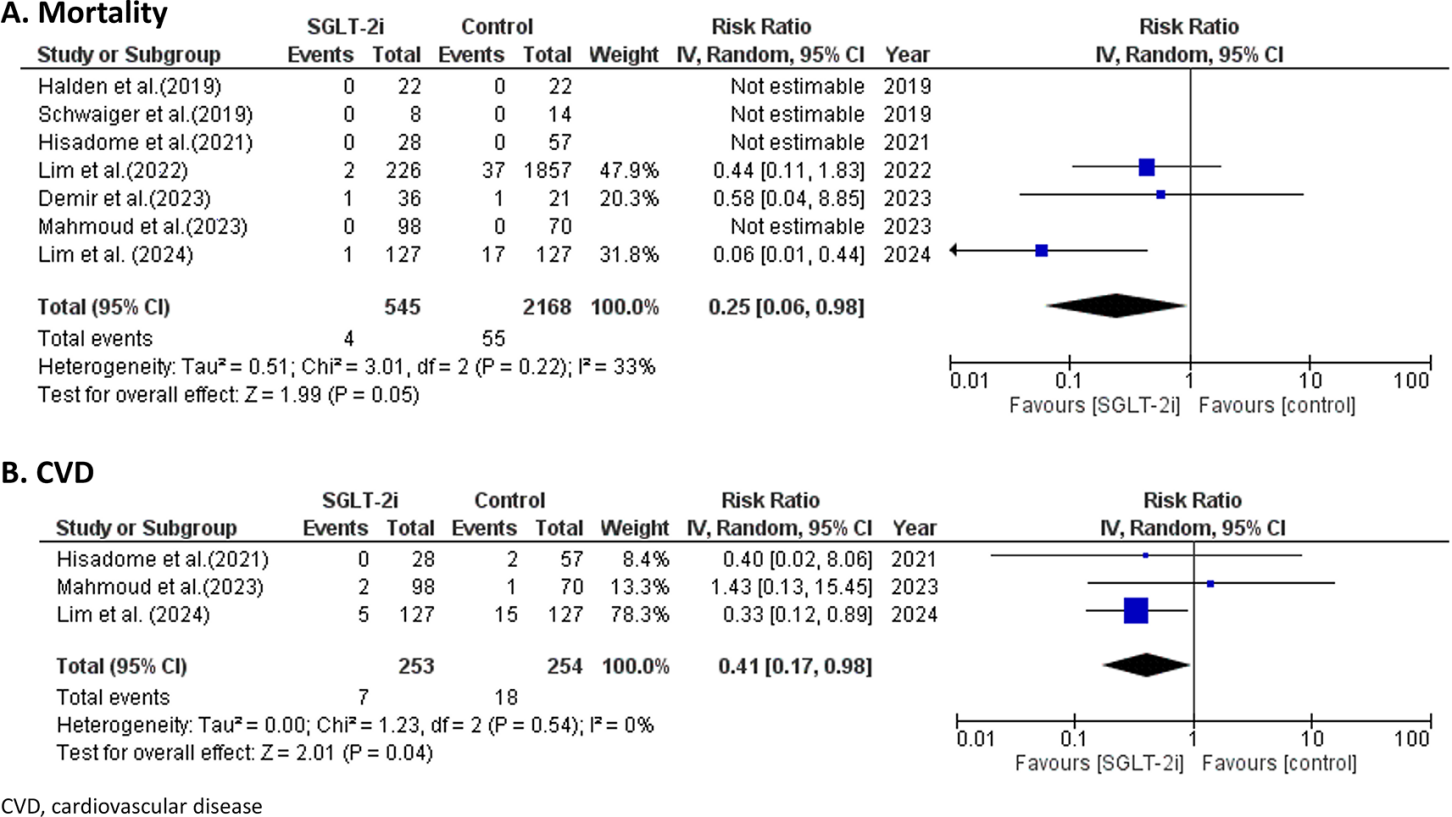
Forest plots of the efficacy of SGLT-2 inhibitors on mortality and CVD.

Among the 7 studies involving 2,168 patients, there were only 40 deaths reported, primarily in 3 studies. ^17, 19, 21^ Over a 9-month follow-up period, SGLT-2 inhibitors showed significantly reduced all-cause mortality compared to placebo, with a risk ratio (RR) of 0.25 (95%CI 0.06, 0.98; p = 0.05; I ^2^ = 33%) as shown in Figure 4A.

Regarding CV outcomes only 3 studies ^18, 20, 21^ involving 253 patients assessed the impact of SGLT-2 inhibitors on cardiovascular disease (CVD). Over a follow-up period of 9 months, SGLT-2 inhibitors showed significantly reduced CVD compared to the control, with a risk ratio (RR) of 0.41 (95%CI 0.17, 0.98; p = 0.04; I ^2^ = 0%) as shown in Figure 4B.

### Adverse events of SGLT-2 inhibitors

**Figure 5.**
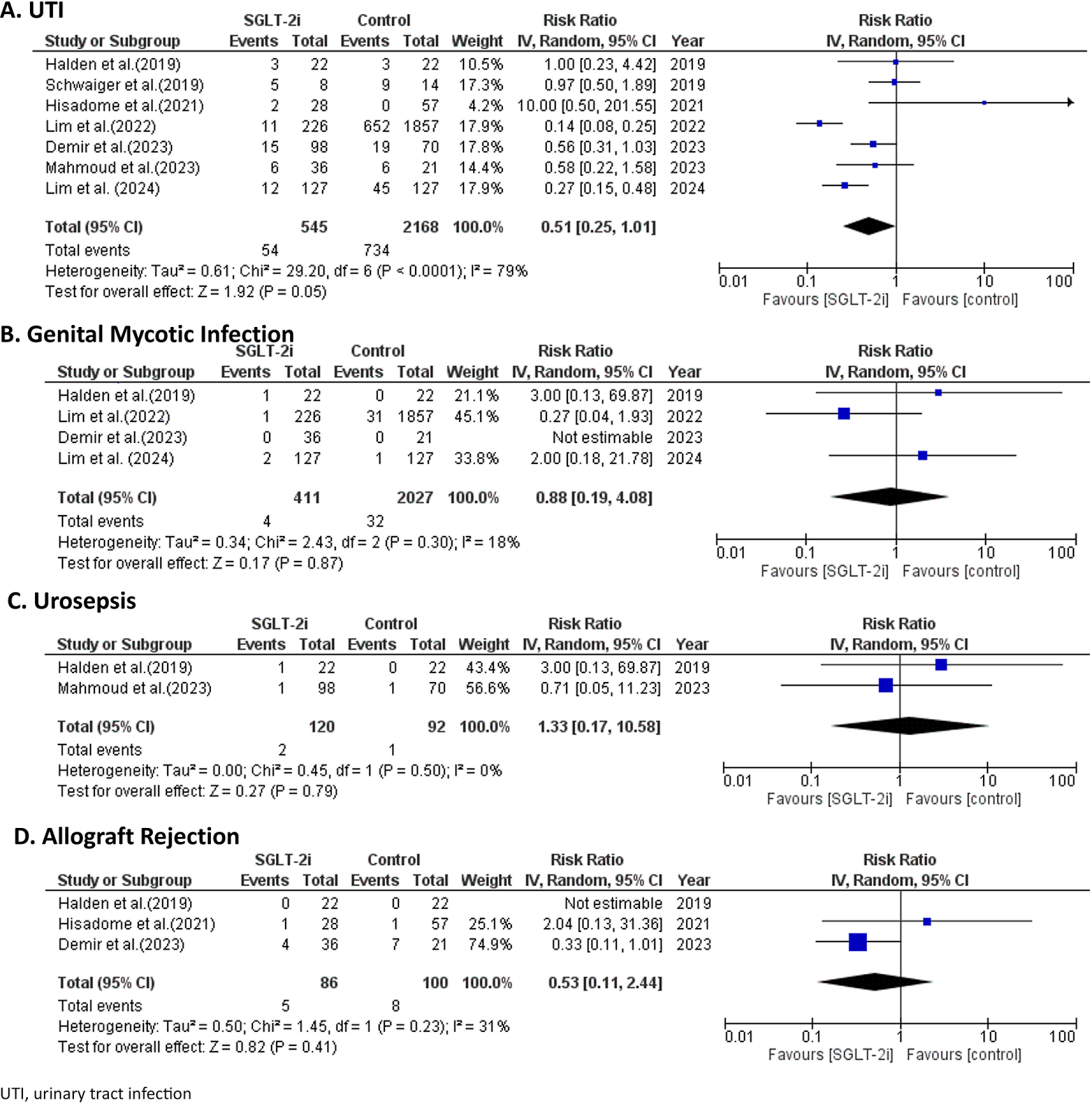
Forest plots of the adverse events of SGLT-2 inhibitors.

There were no significant differences in adverse events incidence between the SGLT-2 inhibitors and the control group. In terms of infection, SGLT-2 inhibitors did not significantly increase the risk of overall urinary tract infection (UTI), compared to the control group, with a RR of 0.51 (95% CI 0.25, 1.01; p = 0.05) with a significant heterogeneity (I ^2^ = 79%) across 7 studies ^17– 23^ as shown in Figure 5A. When specifically examining genital mycotic infections, only 4 studies with 2,438 patients reported outcomes. ^17, 19, 21, 22^ SGLT-2 inhibitors also did not significantly increase the risk of genital mycotic infection compared to the control, with a RR of 0.88 (95% CI 0.19, 4.08; p = 0.87; I ^2^ = 18%) as shown in Figure 5B. Of 2 studies ^20, 22^ involving 212 patients that evaluated the outcome of urosepsis, SGLT-2 inhibitors also did not significantly increase the risk of urosepsis, with a RR of 1.33 (95% CI 0.17, 10.58; p = 0.79; I ^2^ = 0%) as shown in Figure 5C.

Regarding other adverse events, SGLT-2 inhibitors did not significantly increase the risk of allograft rejection compared to the control, with a RR of 0.53 (95% CI 0.11, 2.44; p = 0.23; I ^2^ = 31%) across 3 studies, ^17, 18, 22^ involving 186 patients as shown in Figure 5D. There were no any diabetic ketoacidosis (DKA) cases reported among included studies.

### Evaluation of publication bias

The funnel plots depicting standard error by mean differences and risk ratios were evaluated, as illustrated in Supplemental Figure S5-S7 (Refer Extended data). These assessments revealed no significant evidence of publication bias, as the plots exhibit symmetry.

## Discussion

Our systematic review and meta-analysis focused on the safety and efficacy of SGLT-2 inhibitors among KTRs. Our findings indicate that SGLT-2 inhibitors significantly reduced HbA1c and BMI compared to the control, without increasing adverse events, including infection and allograft rejection. Notably, while SGLT-2 inhibitors showed no measurable impact on kidney-related outcomes, they conferred a substantial benefit by markedly reducing mortality and cardiovascular disease risk among KTRs.

In non-transplant population, a recent meta-analysis of large placebo-controlled trials demonstrated a significant 37% reduction in the kidney disease progression among T2 DM patients using SGLT-2 inhibitors. ^24^ This kidney benefit was consistent across subgroups, regardless of diabetic status, primary kidney diseases, or kidney function. ^24^ The renal benefits of SGLT-2 inhibitors can be attributed to increased urinary glucose excretion, leading to osmotic diuresis and enhanced excretion of sodium and fluid. ^25^ This helps mitigate glomerular hyperfiltration, reduce renal inflammation and fibrosis, and preserve renal function. ^25^ Based on these findings, similar renal benefits may be anticipated in the context of kidney transplant recipients (KTRs). However, the mechanism of SGLT-2 inhibitors, which promotes glucose excretion via urine, could potentially increase infection risk—an important concern in this population.

In contrast to findings in the non-transplant population, our meta-analysis revealed a neutral impact of SGLT-2 inhibitors on kidney function, as assessed by eGFR and UPCR. This finding was consistent with a previous meta-analysis by Chewcharat et al, ^13^ which demonstrated no significant decline in eGFR and UPCR by the end of the study with SGLT-2 inhibitors. This neutral effect may be attributed to the comparatively shorter follow-up periods, with a median duration of 12 months. In contrast, non-transplant studies typical have 2-3 years follow-ups, during which the renal benefits of SGLT-2 inhibitors, especially in eGFR, become more pronounced compared to the placebo group after 12 months. ^26– 28^

Given that KTRs have a significant elevated risk of CV death, 10 to 20 times higher than the general population, ^5, 29^ SGLT-2 inhibitors have been proposed as a new hope in this group. Previous meta-analysis in non-transplant CKD patients have shown promising results for SGLT-2 inhibitors in reducing composite CV death or hospitalization for heart failure, with a remarkable 23% decrease compared to placebo. These finding were observed in patients with an average baseline eGFR of 65 mL/min/1.73 m ^2. 24^ The recent EMPA-KIDNEY trial, ^26^ and DAPA-CKD trial, ^27^ which included CKD patients with eGFR as low as 20 and 25 mL/min/1.73 m ^2^, respectively, demonstrated a significant reduction in CV death: 16% with empagliflozin and 31% with dapagliflozin over a 2-year follow-up period. This benefit was consistent across a range of eGFR levels, though KTRs were not included in these trials. Despite the absence of large-scale studies specifically involving KTRs, our meta-analysis reveals that SGLT-2 inhibitors significantly reduce mortality and cardiovascular disease in this unique population, offering a compelling new therapeutic avenue to address their elevated cardiovascular risk.

Regarding the efficacy of SGLT-2 inhibitors on metabolic profiles, the reduction of 0.37% in HbA1c levels compared to the control group aligns with previous meta-analyses conducted by Okinomaki et al. ^30^ and Chewcharat et al., ^13^ which reported reductions of 0.45% and 0.57% in HbA1c levels from baseline, respectively. Our findings demonstrated that SGLT-2 inhibitors are also effective when compared to standard therapies, including insulin and other oral hypoglycemic agents. Given that tacrolimus can elevate blood glucose levels by inducing insulin resistance and increasing glucose absorption in the gastrointestinal tract, ^31^ it should be noted that 82.5% of our patients received tacrolimus as part of their immunosuppressive regimen. Moreover, our meta-analysis showed that SGLT-2 inhibitors also benefit BMI, with a significant reduction of 0.89 kg/m ^2^ compared to the control. This is consistent with the previous 2 meta-analyses conducted in non-diabetic adults with overweight, which reported a reduction of 0.47 kg/m ^2^ compared to placebo. ^32, 33^

Within current clinical practice, concerns persist regarding the potential adverse events associated with SGLT-2 inhibitors, particularly UTIs ^13^ and DKA, ^34^ which may pose a greater risk for KTRs due to their concurrent administration of immunosuppressive agents. ^35^ However, our meta-analysis observed that SGLT-2 inhibitors did not increase risk of serious infection, including UTIs, genital mycotic infection, and urosepsis, compared to the control group. Notably, a meta-analysis conducted by Li et al., ^36^ evaluating UTIs and genital infections in non-transplant T2DM patients, revealed that only dapagliflozin 10 mg exhibited a significantly higher incidence of UTIs than placebo. In our analysis, only 20.6% of cases was prescribed with dapagliflozin. The overall infection rate of SGLT-2 inhibitors was comparable to that of the control group. Importantly, our study did not find an increase rate of genital infection with SGLT-2 inhibitors, unlike in non-transplant population. ^36^ This may be attributed to the effective routine post-transplant screening, care, and patient education in transplant clinics. Additionally, our meta-analysis demonstrated that SGLT-2 inhibitors did not increase the risk of DKA and graft rejection. Therefore, our findings shed new light on the safety profile of SGLT-2 inhibitors in kidney transplant recipients.

To the best of our knowledge, our meta-analysis included the most recent studies investigating the efficacy and safety outcomes of SGLT-2 inhibitors in KTRs. This encompasses both preexisting T2DM and PTDM status and is the first to assess mortality and cardiovascular outcomes, which was absent from previous meta-analysis. ^13^ However, certain limitations must be acknowledged. Firstly, there are limited studies in our systematic review due to the lack of current studies in KTRs populations, which most existing studies are case series or observational designs without control groups. Secondly, there was significant heterogeneity among included studies, particularly in HbA1c, SBP, and UTI outcomes. This may result from the limited number of studies and the different types of studies for each outcome. Thirdly, the subgroup analysis based on study types (clinical trial vs cohort) and DM type (T2DM vs PTDM) could not be performed due to the limited available data in the included studies. Fourthly, the funnel plots for publication bias need to be interpreted with caution due to the limited number of included studies. Lastly, the follow-up duration in included studies might not have been sufficiently long to fully elucidate the potential renal benefits with SGLT-2 inhibitors.

## Conclusion

SGLT-2 inhibitors have demonstrated significant benefits in reducing mortality and cardiovascular disease in diabetes KTRs. Furthermore, they effectively reduced HbA1c and BMI compared to controls, without increasing adverse events, including infections and allograft rejection. However, these agents did not show a significant benefit in kidney-related outcomes.

## Ethical considerations

This study was approved by the Human Research Ethics Committee of Thammasat University (Medicine), which provided an exemption for this study (COE 243/2567).

## Reporting guidelines

Zenodo: The Efficacy and Safety of SGLT2 Inhibitors in Diabetes Kidney Transplant Recipients: A Systematic Review and Meta-Analysis-supplemental materials and PRISMA checklist, DOI: https://doi.org/10.5281/zenodo.14928362. ^37^

The project contains the following reporting guidelines:

• PRISMA checklist
• Flow chart

Data are available under the terms of the Creative Commons Attribution 4.0 International license (CC-BY 4.0).

## Data Availability

No data are associated with this article. Zenodo: The Efficacy and Safety of SGLT2 Inhibitors in Diabetes Kidney Transplant Recipients: A Systematic Review and Meta-Analysis-supplemental materials and PRISMA checklist, DOI: https://doi.org/10.5281/zenodo.14988603. ^37^ This project contains the following extended data:
•
Table S1 Search Terms •
Table S2 Risk of bias summary for included RCT •
Table S3 Graph and summary of Risk of Bias in Non-randomized Studies of Interventions (ROBIN-I) for included non-randomized controlled studies •
Figure S4 Funnel Plots of Standard Error on Kidney-Related Outcomes •
Figure S5 Funnel Plots of Standard Error on Metabolic Profiles •
Figure S6 Funnel Plots of Standard Error on Mortality and Cardiovascular Diseases •
Figure S7 Funnel Plots of Standard Error on Adverse Events Data are available under the terms of the Creative Commons Attribution 4.0 International license (CC-BY 4.0).
