# Supplementary material for: The Efficacy and Safety of SGLT2 Inhibitors in Diabetes Kidney Transplant Recipients: A Systematic Review and Meta-Analysis
Source: F1000Res. 2025 Mar 25;14:329. [Version 1] doi: 10.12688/f1000research.162502.1 (PMC12739370; doi:10.12688/f1000research.162502.1)
Supplement: Peer review history

# Reviewer report: Reviewer response for version 1

**DOI:** 10.5256/f1000research.178712.r432370

**Published:** 2025-12-24

**Authors:** Yang Xiao

**Affiliations:** 1 Central South University, Changsha, China

The rationale is explicitly presented.The study provides comprehensive methodological details.The statistical approach aligns with standard meta-analysis practices. The conclusions directly reflect the study results.But,the title and abstract do not mention “living” terminology，The search was conducted up to a fixed date.The study’s major significance lies in addressing an unmet clinical need: evaluating SGLT-2 inhibitors in diabetic KTRs.But,when an abbreviation is first used, its full form must be provided ."IPTW" is not explained in Table 1And in the discussion, a reasonable explanation should be provided for the "kidney results".

Are the rationale for, and objectives of, the Systematic Review clearly stated?

Yes

Is the statistical analysis and its interpretation appropriate?

Yes

If this is a Living Systematic Review, is the ‘living’ method appropriate and is the search schedule clearly defined and justified? (‘Living Systematic Review’ or a variation of this term should be included in the title.)

No

Are sufficient details of the methods and analysis provided to allow replication by others?

Yes

Are the conclusions drawn adequately supported by the results presented in the review?

Yes

Reviewer Expertise:

metabolic diseases，immunometabolism，diabetes

I confirm that I have read this submission and believe that I have an appropriate level of expertise to confirm that it is of an acceptable scientific standard.

# Reviewer report: Reviewer response for version 1

**DOI:** 10.5256/f1000research.178712.r432374

**Published:** 2025-12-23

**Authors:** Shatavisa Mukherjee

**Affiliations:** 1 Calcutta School of Tropical Medicine, Kolkata, West Bengal, India

This manuscript does not meet the scientific, statistical, or interpretive standards required for publication as a meta-analysis and should be rejected in its current form. Although the topic is clinically relevant, the analysis is fundamentally flawed and the conclusions are misleading. The authors rely predominantly on small retrospective observational cohorts with heterogeneous comparator groups and inadequate adjustment for confounding, yet present pooled mortality and cardiovascular outcomes as though derived from robust randomized evidence. The mortality signal, based on only 40 events from three studies with short follow-up, is statistically fragile and unsuitable for causal inference, particularly in a transplant population where competing risks and selection bias are profound. Likewise, cardiovascular benefit is asserted from fewer than 300 patients across three cohorts, representing an insufficient evidential base for clinical recommendations.

 A major methodological error is the inappropriate use of a random-effects model as a default analytic strategy. While random-effects modeling is theoretically justified when studies estimate different underlying effects, it is not a solution for combining fundamentally incomparable designs and exposures. In this manuscript, randomized trials, uncontrolled interventional studies, and retrospective cohorts are improperly pooled; more critically, control arms ranging from placebo to insulin, metformin, sulfonylureas, and DPP-4 inhibitors are treated as if they were equivalent. Under these conditions, random-effects modeling merely averages bias rather than accounts for heterogeneity. This issue is magnified in outcomes with sparse events, where random-effects weighting disproportionately favors small, unstable cohorts and can artificially amplify significance. The authors neither justify model choice nor conduct influence analysis, leave-one-out testing, or meta-regression to explore extreme observed heterogeneity (e.g., HbA1c I² > 90%), instead defaulting to random-effects without methodological rationale. The result is spurious numerical precision with little biological or clinical meaning.

 The renal analysis—the central justification for transplant relevance—is underpowered and inconclusive. Proteinuria outcomes are based on fewer than 200 patients, and eGFR analyses show substantial heterogeneity with no detectable effect. Despite this, mechanistic speculation on kidney protection is presented in the discussion without supporting evidence. Safety conclusions are equally unreliable: infection, rejection, and ketoacidosis outcomes are derived from short-term datasets with very wide confidence intervals in an immunosuppressed population, yet the authors assert clinical safety based on non-significant findings—a classic misinterpretation of statistical non-difference as proof of absence of harm.

 Statistical rigor is notably lacking. Multiplicity across many secondary endpoints is ignored. Funnel plots are used inappropriately in a dataset with too few studies to meaningfully assess publication bias. Heterogeneity is reported but not interrogated. Planned subgroup analyses were abandoned without analytical restructuring. The manuscript repeatedly imports mechanistic and outcome evidence from non-transplant populations to support conclusions in kidney transplant recipients, ignoring the unique pharmacology, immunosuppressive interactions, and metabolic profiles of this cohort.

 The Discussion and Conclusions substantially overstate the evidence, with language implying disease-modifying, cardioprotective, and mortality-reducing effects that are not supported by the data. The recurring framing of SGLT-2 inhibitors as a “therapeutic breakthrough” in kidney transplantation is unsupported and risks misleading readers into inappropriate extrapolation. At best, the current evidence supports cautious short-term glycemic and weight effects; it does not justify claims of cardiovascular protection, safety assurance, or survival benefit in this population.

 This is not a failure of reporting but of design, methodology, and interpretation. Without study-level stratification, comparator-specific analyses, conservative inference, and elimination of causal language, this work does not meaningfully advance knowledge and carries a significant risk of clinical misinterpretation.

Are the rationale for, and objectives of, the Systematic Review clearly stated?

Partly

Is the statistical analysis and its interpretation appropriate?

No

If this is a Living Systematic Review, is the ‘living’ method appropriate and is the search schedule clearly defined and justified? (‘Living Systematic Review’ or a variation of this term should be included in the title.)

Partly

Are sufficient details of the methods and analysis provided to allow replication by others?

Partly

Are the conclusions drawn adequately supported by the results presented in the review?

No

Reviewer Expertise:

Pharmacoepidemiology, Pharmacology, Biostatistics, Research Ethics

I confirm that I have read this submission and believe that I have an appropriate level of expertise to state that I do not consider it to be of an acceptable scientific standard, for reasons outlined above.

# Reviewer report: Reviewer response for version 1

**DOI:** 10.5256/f1000research.178712.r378428

**Published:** 2025-05-12

**Authors:** Tarek Mahmoud

**Affiliations:** 1 Jaber Al Ahmed Armed Forces Hospital, Subhan, Kuwait

**Article Overview**

 This study aimed to evaluate the efficacy and safety of sodium-glucose co-transporter 2 (SGLT2) inhibitors in kidney transplant recipients (KTRs) with diabetes. After comprehensive literature search including observational and clinical trials they collected seven studies. There results indicated that SGLT2 inhibitors effectively reduced mortality, cardiovascular disease, HbA1c and BMI without increasing risk of infections and allograft rejection however it did not show clear benefit in kidney outcomes.

**Strengths**

- The systematic review and meta-analysis helped to gather data randomly from the related studies reducing the bias and strengthening the findings.
- The improvement in diabetes related and cardiovascular end-points supported the effectiveness of SGLT2 inhibitors in managing diabetes in KTRs.
- The absence of serious adverse events, specifically the urinary tract and genital infections, supports the safety of SGLT2 inhibitors in this population.

**Limitations**

- The majority of the included studies were case series and not randomized controlled ones, which is understandable from the available studies in the literature, but this may introduce bias and limit the generalizability of the results.
- The criteria for selection may be tight to allow some more studies, specially early ones, to be included.
- The selected studies have different designs and inclusion criteria which makes the comparison difficult.
- The median follow-up period was 9 (9,12) months, which may not be enough to assess renal outcomes and detect long-term complications or benefits.​

**Clinical Implications**

             The study results support the benefits of SGLT2 inhibitors in managing diabetic KTRs with reasonable safety. However, the limitations highlight the need of larger randomized controlled trials including also non-diabetic KTRs with longer follow up duration to confirm these findings and lay down the guidelines for their use.

**Conclusion**

 This systematic review and meta-analysis will add to the knowledge on the use of SGLT2i for KTRs. It should also stimulate further research to validate these findings and establish a comprehensive treatment guidelines.

 N.B. in the title " ... *Diabetes* Kidney Transplant Recipients" It should be " ... *Diabetic* ..."

Are the rationale for, and objectives of, the Systematic Review clearly stated?

Yes

Is the statistical analysis and its interpretation appropriate?

I cannot comment. A qualified statistician is required.

If this is a Living Systematic Review, is the ‘living’ method appropriate and is the search schedule clearly defined and justified? (‘Living Systematic Review’ or a variation of this term should be included in the title.)

Yes

Are sufficient details of the methods and analysis provided to allow replication by others?

Yes

Are the conclusions drawn adequately supported by the results presented in the review?

Yes

Reviewer Expertise:

Kidney transplantation, outcomes in special groups, diabetes, transplant infections, pregnancy, Ramadan Fast.

I confirm that I have read this submission and believe that I have an appropriate level of expertise to confirm that it is of an acceptable scientific standard.
